# Supplementary material for: Supporting self-management for patients with Interstitial Lung Diseases: Utility and acceptability of digital devices
Source: PLOS Digit Health. 2024 Jan 8;3(1):e0000318. doi: 10.1371/journal.pdig.0000318 (PMC10773949; doi:10.1371/journal.pdig.0000318)
Supplement: Attachment — Submitted filename: Response to Reviewers.docx [file pdig.0000318.s002.docx]

**A point-by-point response to the reviewer’s comments**

**Dear Editor,**

Thank you for the opportunity to resubmit our revised survey entitled “Supporting self-management for patients with Interstitial Lung Diseases: Utility and Acceptability of Digital Devices.” for consideration for publication in the prestigious PLOS Digital Health. We are grateful to you and the reviewer’s insightful comments on our paper, and we confirm that we have revised our paper in accordance with your suggestions.

Thank you so much for your kind consideration.

Sincerely,

**Anne-Marie Russell**

**Dr AM Russell**

**University of Exeter**

**On behalf of the authors.**

**Journal Requirements:**

**1. Please send a completed 'Competing Interests' statement, including any COIs declared by your co-authors. If you have no competing interests to declare, please state "The authors have declared that no competing interests exist". Otherwise please declare all competing interests beginning with the statement "I have read the journal's policy and the authors of this manuscript have the following competing interests:"**

*We have read the journal's policy, please see below – we will submit all* ICMJE disclosure form*s*

**2. In the Funding Information you indicated that no funding was received. Please revise the Funding Information field to reflect funding received.**

*RS was supported by a Health Education England Population Health fellowship. The funders had no role in study design, data collection and analysis, decision to publish, or preparation of the manuscript. Funder’s URL:* [*https://www.hee.nhs.uk/our-work/population-health/population-health-fellowship-0*](https://www.hee.nhs.uk/our-work/population-health/population-health-fellowship-0)

3. Please send a completed 'Competing Interests' statement, including any COIs declared by your co-authors. If you have no competing interests to declare, please state "The authors have declared that no competing interests exist". Otherwise please declare all competing interests beginning with the statement "I have read the journal's policy and the authors of this manuscript have the following competing interests:"

*AMR reports grants, personal fees and other from Boehringer Ingelheim , personal fees from Hoffman La Roche, outside the submitted work. AMR is a NIHR Senior Research Leader. The views expressed in this publication are those of the author(s) and not necessarily those of the National Institute for Health Research or the Department of Health and Social Care.*

*MAG and SL have received travel grants and honoraria from Boehringer Ingelheim for speaking / participation at meetings outside the submitted work JL has received research grants from GSK and Astra Zeneca outside the submitted work*

*We have added this statement to the manuscript and will send completed ICMJE DISCLOSURE FORMs via email or upload to the portal.*

**4. Please amend your detailed Financial Disclosure statement. This is published with the article. It must therefore be completed in full sentences and contain the exact wording you wish to be published.**

**a. State the initials, alongside each funding source, of each author to receive each grant.**

Thank you – we have attended to this

**b. State what role the funders took in the study. If the funders had no role in your study, please state: “The funders had no role in study design, data collection and analysis, decision to publish, or preparation of the manuscript.”**

*The funders had no role in study design, data collection and analysis, decision to publish, or preparation of the manuscript.*

**c. If any authors received a salary from any of your funders, please state which authors and which funders.**

*The authors received no specific funding for this work.*

**5. Please provide separate figure files in .tif or .eps format only and remove any figures embedded in your manuscript file. Please also ensure that all files are under our size limit of 10MB.**
*We have provided the required figure files in.tif format, and ensured that each file is under the 10MB size limit. The figures previously embedded within the manuscript have been removed.*

**6. We ask that a manuscript source file is provided at Revision. Please upload your manuscript file as a .doc, .docx, .rtf or .tex.**

*We have uploaded our revised manuscript as docx file to meet this requirement.*

7. In the online submission form, you indicated that "Analysis was done in Excel and NVIVO - Raw anonymised data is available on direct request to the corresponding author for legitimate non-commercial purposes". All PLOS journals now require all data underlying the findings described in their manuscript to be freely available to other researchers, either 1. In a public repository, 2. Within the manuscript itself, or 3. Uploaded as supplementary information.
This policy applies to all data except where public deposition would breach compliance with the protocol approved by your research ethics board. If your data cannot be made publicly available for ethical or legal reasons (e.g., public availability would compromise patient privacy), please explain your reasons by return email and your exemption request will be escalated to the editor for approval. Your exemption request will be handled independently and will not hold up the peer review process, but will need to be resolved should your manuscript be accepted for publication. One of the Editorial team will then be in touch if there are any issues.

*Thank you we are looking into this and will revert asap*

**Additional Editor Comments (if provided):**

The manuscript was interesting. Please consider the following comments in your revision.

**1- Please follow the journal style for formatting the abstract and the manuscript.**

*We understand the importance of adhering to the journal's style guide for formatting the abstract and manuscript. In our revision, we confirm that we followed the formatting guidelines provided by the journal.*

**2- In the methods section, please add adequate information about the study participants and sampling methodology.**

*We appreciate your suggestion to provide more detailed information on the study participants and the sampling methodology in the Methods section. We have revised this section, and included comprehensive information to explain how participants were included, the criteria for inclusion and exclusion, and the techniques used for sampling. P1 abstract:*

*“A team of expert clinicians and patient partners interested in self-management approaches collaboratively designed a 48-question cross-sectional electronic survey. The survey was specifically targeted at individuals diagnosed with ILD. The survey was critically appraised by the interdisciplinary self-management group at Royal Devon University Hospitals NHS Foundation Trust during a 6-month validation process. The survey was open for participation between September 2021 and December 2022, and responses were collected anonymously. Data were analysed descriptively for quantitative aspects and through thematic analysis for qualitative input. ”*

**Reviewers' comments:**

**Reviewer #1:**

**General**
**1.There are capitalization errors, punctuation errors, missing symbols (trademark or copyright, such as for commercial software and technology) and run-on sentences throughout (not further identified in this review)**

*We sincerely apologise for these errors and any confusion they may have caused. To address these issues, we have carefully reviewed and revised the entire manuscript to correct these mistakes. The text has been thoroughly edited for language, consistency, and adherence to journal guidelines, including the proper use of trademark and copyright symbols where appropriate. We appreciate your attention to detail, which helps improve the quality and readability of our paper.*

**2. While the purpose is identified, the abstract lacks sufficient details regarding clear statement of study objectives, I/E criteria for respondents, survey tool validation, timeline, data areas/elements, content areas, and data analysis (descriptive, hypothesis testing, etc).  It is unclear if the responses were anonymous.  Without these elements, the data summary appears to be somewhat random, and it is unclear if the conclusions address the objectives and/or are justified.**

We acknowledge the lack of detail in the abstract concerning the statement of objectives, I/E criteria, survey tool validation, timeline, and data analysis. We have revised the abstract to include these elements.

*Objectives: We sought to quantify patients' usage and experiences with home digital devices, and further evaluate their perceived utility and barriers to adaptation.*

*Methods: A team of expert clinicians and patient partners interested in self-management approaches collaboratively designed a 48-question cross-sectional electronic survey. The survey was specifically targeted at individuals diagnosed with ILD. The survey was critically appraised by the interdisciplinary self-management group at Royal Devon University Hospitals NHS Foundation Trust during a 6-month validation process. The survey was open for participation between September 2021 and December 2022, and responses were collected anonymously. Data were analysed descriptively for quantitative aspects and through thematic analysis for qualitative input.*

**3. Abstract (and elsewhere) states 65 respondents completed the survey but reports data on the 89 that met eligibility criteria.  It is therefore unclear if survey completion is needed for data inclusion. The denominator for responses is inconsistent, implying incomplete survey or data subsets being presented. Because of the changing denominators, they should be consistently reported throughout the results section as well.**

*We apologise for any confusion this may have caused. To clarify, the number '89' represents those who met the eligibility criteria and started the survey, whereas '65' represent those who completed the survey in full. Data from partially completed surveys were also included in the analysis, as we aimed to capture as much information as possible. We understand that this creates variability in the denominators for individual questions, which we have clarified in the methods that 'We present data by the number of respondents and percentage proportionate to the total number of responses per item.' – page 5*

**4. Much of the data summarized in the results narrative is available in summary form elsewhere and is difficult to follow.  Demographics could best be summarized by a table.**

*We agree that a summary table for demographics would enhance readability and comprehension. We have revised the results section to include the demographics variables Table 1.*

| Table 1. Demographics variables | % | Frequency |
| --- | --- | --- |
| Gender |  |  |
| Male | 33.7% | 30 |
| Female | 66.3% | 59 |
| Continent |  |  |
| Europe - UK | 80.0% | 52 |
| USA | 1.5% | 1 |
| Canada | 4.6% | 3 |
| Asia | 13.8% | 9 |
| UK Region |  |  |
| London and home counties | 11.5% | 6 |
| Southeast | 13.5% | 7 |
| Southwest | 26.9% | 14 |
| Midlands | 26.9% | 14 |
| Northwest | 3.8% | 2 |
| Scotland and Highlands | 11.5% | 6 |
| Northern Ireland | 1.9% | 1 |
| Other | 3.8% | 2 |
| Age |  |  |
| 18 to 40 | 6.7% | 6 |
| 41-50 | 10.1% | 9 |
| 51 – 60 | 19.1% | 17 |
| 61 – 70 | 29..2% | 26 |
| 71 -80 | 23.6% | 21 |
| 80 – 85 | 10.1% | 9 |
| >85 | 1.1% | 1 |

**5. Given the variable denominators for responses, it is essential to indicate the total number of responses within a category so that the percentages can be verified.  In some cases, data within a topic area with what appears to be exclusive responses totals more than 100%.**

*We understand the need for clarity regarding variable denominators. We have ensured that the total number of responses within each category is clearly indicated, allowing for percentages to be verified.*

**Specific
1. (p2) reference to “….. device issue and data collection” is out of context and unclear. No description of what devices/technology are being utilized by patients and included as a target by this study in the Introduction section.**

*We have elaborated on what devices or technology are being targeted in this study in the Introduction section.*

*Digital devices are commonly suggested for all individuals with ILD in order to improve their ability to manage and monitor their condition. However, the importance of these devices is most evident for those who experience long-term breathlessness and psychological discomfort [6]. Home monitoring via commercially available devices that measure physiological parameters and symptoms may provide clinicians and patients with access to more precise continuous data on disease progression, the rate of acute exacerbations, and effects on quality of life [6-11]. This enables the development of personalised treatment approaches in this cohort [6, 12]. Supported self-management measures monitor the progression of a patient’s condition objectively, with digital technologies and subjectively, through the completion of patient-reported measures capturing symptom experiences and health-related quality-of-life (HRQOL) [6, 8]. The use of digital devices, in this context, aligns with the need for early intervention and cost-effective management, reinforcing their relevance and utility for patients with ILD [6, 8, 13].*

**2. (p2) what is meant by “….across the wider i healthcare team.”?**

We apologise for the ambiguity. We have updated the sentence as follow:

*This approach requires support from the dedicated interdisciplinary ILD team with effective and efficient communications across the wider interdisciplinary healthcare team including primary care [14]. The National Institute for Health and Care Excellence (NICE) guidelines (2017), recommended that the minimally required members of an ILD team include a physician, radiologist, specialist nurse, and MDT coordinator. In more complex diagnostic or treatment situations, the team should also involve thoracic surgeons, histopathologists, rheumatologists, occupational physicians, and geneticists [15].*

**3.(p3) for survey content area, what is the difference between “device” and “technology”.**

*Thank you for bringing this to our attention. To clarify, we have updated the manuscript to consistently use 'digital devices' instead of 'technology' to better focus the survey content and eliminate any ambiguity.*

**4. (p3) there is no clear description within the methods section of subject inclusion/exclusion, response inclusion/exclusion (such as the need for completed/partial responses), timeline (dates), geographic distribution**

*We have included clear descriptions of the inclusion/exclusion criteria, the types of responses needed, the timeline, and the geographic distribution of the survey in the Methods section.*

*“The brief clearly stated that the survey was specifically for people diagnosed with ILD; those who were not diagnosed with ILD were consequently excluded from the study. This international survey was open for participation from September 2021 to January 2022. It was anonymous with clear information on how data collected would be used.” Page 5*

**5 (p5) Qualitative data summaries using descriptions like “several participants…” and “some participants” might be confusing to many readers.**

*Thank you - We have replaced and quantified these terms.*

**6 (p6, Table 1). Clarify total number of responses so that % can be calculated for each response. Yes/No answer can be consolidated into “Use of device to monitor vital signs (n, %).  Its unclear if device type categories should be exclusive for the first 3 choices, yet total more than 100%.  Unclear how branching logic impacted %, since those not using devices could not respond to type and advice.  % Totals within categories (such as Advise on interpreting spirometry readings) with exclusive responses exceed 100%.**

*We have revised the table to address these issues you have raised.*

| *Survey variables and responses* | *%* | *F* |
| --- | --- | --- |
| *Uses devices to monitor vital signs* | *86%* | *76* |
| *Does not use devices to monitor vital signs* | *14%* | *12* |

**7 (p8) Statement beginning “Whilst we recommend….” Is confusing and should be reworded.  What is meant by “CE marked”?**

*The term "CE marked" refers to products that meet European safety, health, and environmental protection requirements. We have rephrased the statement for clarity.*

**8 (p10) Denominators for the responses are not provided. Uncertain why ID numbers and genders are provided within the narrative.**

*We have included the denominators for responses and removed irrelevant information where possible, such as ID numbers and genders. However, given the gender differences across ILDs and an interest in the community to expanding programmes undertaken in IPF for example to CTD ILD (male dominant to female dominant) we think that this is of interest to the readership*

**9 (Figure 1) Contains overlapping data quantity labels within the figure so unable to interpret. No figure legend was provided.**

*We have revised the figure to eliminate overlapping data labels and provide a figure legend for clarity.*

**10. (Figure 2) Lacks quantitative data to determine how frequent the themes were presented. Perhaps this could be done by the size of the theme’s figure. A figure legend is needed to explain the connections.**

*Thank you for your comment about the lack of quantitative data in Figure 2. We appreciate your suggestion to make the frequency of themes more explicit. Whilst in political campaigns word counts are frequently used it is not the accepted approach for thematic qualitative analysis – we have identified overarching and sub themes and made this more explicit in the body of the text. We have also updated the figure legend to further explain these connections and to provide a clearer representation of each theme.*

**11.(Figure 3) see above comments re: Figure 2**

We have updated figure 3 legend to further explain these connections and to provide a clearer representation of the themes.

12. (Figure 4) not included in my copy

*We apologise for this. We have ensured that Figure 4 is included in the revised manuscript*

.

Reviewer #2:

**In the introduction, page 5, the authors indicate that digital devices should be used by ILD patients experiencing intractable breathlessness and psychological distress. The literature indicates that digital devices are recommended for all patients having ILD. It would be good if the authors clarify why they only recommend digital devices in critical conditions. Are digital devices useful in other conditions in ILD patients?**

*We agree that digital devices have applications beyond these specific cases. We revised the introduction to include this broader context and cited this at Q1 in the Specif section above*

There are some typo mistakes that should be corrected.

*We apologise. We have proof read more carefully and hope that we have corrected all typos and apologise for the error*.

The conclusion is very short for the work that the authors present. What are the conclusions about concerns or difficulties experienced by the patients? Any way to address those concerns and difficulties? Any suggestions about the periodicity to use the digital devices? Why?

*Thank you - we understand that the conclusions may appear succinct given the scope of the work. Whilst we do provide and extensive discussion we have expanded this section to address the concerns you raise.*

*‘Our work demonstrates that digital approaches, are an important component of a supported self-management programme but further work is needed to develop bespoke digital pathways of care tailored to individual needs. This requires solution focused thinking to meet the needs of wide ranging digital abilities in those with chronic health conditions and political commitment to optimize wifii connections for those in rural and harder to reach communities, the very people most likely to benefit from supportive self-management approaches. Health economic evaluation of digital care pathways is very much needed alongside an in-depth exploration of the impact of home monitoring using digital devices on the family dynamic. Digital devices will continue to be embedded in our lives and they yield clinically useful data as a component of not a replacement for traditional models of healthcare.*

**The lower right part of figure 3 is practically illegible**.

*We have revised the figure to make sure all parts are legible and will work with the production team on this for maximum quality – we have produced an alternate ‘back-up’ diagram in case of any difficulties*

Reference 8 is from 2023, citation should be corrected.
*We appreciate your attention to detail. The citation was corrected to accurately reflect its publication date.*

“8. NHS NHSNc: COVID Oximetry @home - digital and data services. <https://digital.nhs.uk/coronavirus/covid-oximetry-at-home-digital-and-data-services> (2022). with a recivesed access date ”

**Reviewer #3: This survey study aims to promote the use of digital devices for self-management among patients with interstitial lung disease (ILD). It provides valuable insights into the requirements for enhancing digital device use among ILD patients, a trend that has been growing recently. However, it is necessary to hypothesize why ILD patients need to self-manage using digital devices and the potential benefits their widespread use could bring to healthcare. ILD patients have traditionally been inclined towards self-monitoring and management, such as home oxygen therapy. The assumed benefits of monitoring disease status include early intervention, prevention of disease progression, and reduction in healthcare costs.**

*Thank you for this comment. We have included a section that discusses the various advantages of digital device usage, such as early intervention and detection of disease progression, as shown below.*

*Interstitial Lung Diseases (ILDs) are associated with a symptom burden affecting daily life that is often complex to manage [1]. ILDs may present as multi-system disorders often alongside significant co- morbidities [2, 3].Traditionally, ILD patients have relied on self-monitoring and management methods like home oxygen therapy [4]. However, digital devices can further empower these patients by offering more granular, continuous data which can facilitate early intervention, enable detection of disease progression, and reduce healthcare costs [5, 6].*

*The following paragraph has also been revised and is cited above at Q1*

**The analysis and discussion could be improved, and ideally, the study should be expanded to include more subjects and reassess the following points:**

*We have mentioned this as a limitation and express the need for larger follow-up studies.*

*“The strength of this survey is that it captures both qualitative and quantitative information on remote monitoring in patients with ILD. Nevertheless, the survey has some limitations, for example, the representativeness of the sample, as the participants were predominantly from the UK. The results of this study are consistent with previous research [**41**], but larger and more diverse studies are needed to confirm these findings.”*

**1. Social factors significantly influence the use of digital devices. ILD patients are predominantly older men, often facing income and health literacy challenges. In this study, 33% of patients were under 60 and mostly women, which deviates from the typical ILD patient demographic. It is important to encourage self-management among those not included in this study, particularly those with low digital literacy and self-management difficulties.**

*We acknowledge that our study sample is not entirely reflective of the typical demographic of ILD patients. Also, that patients with different ILDS generally face a different set of challenges that we may not have captured adequately. We have mentioned this as a limitation and express the need for larger follow-up studies and recognise that the distribution method chosen may have contributed to yielding a higher female response*

**2. The type of devices used likely varies based on the patient's disease stage and treatment. It is crucial to analyze the barriers to management and the necessary support, depending on the patient's background and disease stage in each study.**

*We agree that the type of devices used is likely influenced by the patient's disease stage and treatment plan and personal choice – we are undertaking a longitudinal study to identify these issues*

**3. Despite the absence of economic-related questions in the questionnaire, the sudden mention of financial resources and cost-effectiveness issues in the author's eight points on page 18 is concerning. If this is a factor, the questionnaire and analysis should include patient income and perceptions of cost-effectiveness.**

*While it is true that our questionnaire did not explicitly ask about income, we did include questions about financial support and resources for obtaining digital devices. We suspect that digital poverty exists and our work offered a first step to explore how devices are acquired and how this is influenced by society. Our ongoing work is exploring these issues in greater depth. Here we explored the issue of limited resources in Figure 3 of the manuscript. We include the quote from the relevant section for your reference:*

*“Financial support and resources*

*Only 9(12%) reported that their GP or specialist team supplied their devices. 22(32%) reported that they purchased their own informed by online reviews, followed by 12(17%) informed by ILD forum/support groups and 10(14%) on the recommendation of a healthcare professional. An additional 6 (9%) chose their devices because it was the lowest price they could find. The reasons for using health apps and devices had a strong psychological component associated with control 25(40%), as well as a marker of progression 19(30%), and validating feeling unwell 25(40%); (Table 2).”*
